# Melatonin in Early Nutrition: Long-Term Effects on Cardiovascular System

**DOI:** 10.3390/ijms22136809

**Published:** 2021-06-24

**Authors:** Marie Gombert, Pilar Codoñer-Franch

**Affiliations:** 1 Department of Pediatrics, Obstetrics and Gynecology, University of Valencia, 46010 Valencia, Spain; 2 Service of Pediatrics, Hospital Universitario del Doctor Peset, Foundation for the Promotion of Health and Biomedical Research in the Valencian Region (FISABIO), 46010 Valencia, Spain; pilar.codoner@uv.es

**Keywords:** melatonin, breast milk, newborn, early life nutrition, circadian rhythms, antioxidant, oxidative stress, gut microbiota, cardiovascular disorders, cardiovascular programming

## Abstract

Breastfeeding protects against adverse cardiovascular outcomes in the long term. Melatonin is an active molecule that is present in the breast milk produced at night beginning in the first stages of lactation. This indoleamine appears to be a relevant contributor to the benefits of breast milk because it can affect infant health in several ways. The melatonin concentration in breast milk varies in a circadian pattern, making breast milk a chrononutrient. The consumption of melatonin can induce the first circadian stimulation in the infant’s body at an age when his/her own circadian machinery is not functioning yet. This molecule is also a powerful antioxidant with the ability to act on infant cells directly as a scavenger and indirectly by lowering oxidant molecule production and enhancing the antioxidant capacity of the body. Melatonin also participates in regulating inflammation. Furthermore, melatonin can participate in shaping the gut microbiota composition, richness, and variation over time, also modulating which molecules are absorbed by the host. In all these ways, melatonin from breast milk influences weight gain in infants, limiting the development of obesity and comorbidities in the long term, and it can help shape the ideal cellular environment for the development of the infant’s cardiovascular system.

## 1. Introduction

### 1.1. Early Nutrition and Health Programming

The development and subsequent function of virtually all bodily systems have sensitive periods or critical windows of developmental plasticity. Environmental cues perceived during these periods have the capacity to produce significant life-long consequences. This context is the basis for the “developmental origins of health and disease” theory first discussed more than 30 years ago [1].

Early nutrition, including before and after childbirth, during “the 1000-day period,” is one of the most significant environmental determinants of offspring programming in future adults. In addition to fulfilling all the requirements for adequate growth and development with a careful delivery of energy and nutrients, nutrition can influence transcription and genome stability through the epigenetic regulation of gene expression. This epigenetic programming can modify the inflammatory molecular pathways and immune response and even the microbiota with consequences for metabolism, growth, and neurodevelopment in the long term [2].

Naturally provided, human breast milk is a complex and unique fluid that comprises bioactive components necessary for the healthy physical growth, immune system development, and brain maturation occurring during this period [3,4]. Breast milk is also considered the ideal nutrient in early childhood because of its constant adaptation to the evolution of the baby’s needs according to the breastfeeding stage (colostrum, transition milk, and mature milk), in which the available volume, macronutrient composition, and caloric content varies with time and as a function of gestational age at birth. Therefore, exclusive breastfeeding during the first six months of life remains the best way to meet infant nutritional needs [4].

Breast milk as early nutrition is also associated with numerous positive health outcomes, diminishing morbidity and mortality, and protecting against specific processes, such as cardiovascular diseases in adult life, within others [5,6,7]. Evidence suggests that nutrition in early life has profound effects on body weight status. Exclusively breastfed infants show lower rates of infancy weight gain and a lower prevalence of obesity and diabetes, conditions that lead to increasing cardiovascular risk [8,9].

The health benefits of breastfeeding that are classically based on milk components such as lower protein and high lipid content also depend on bioactive molecules, such as hormones involved in the regulation of food intake and amino acids [10], nucleotides [11], immune markers [12], and microRNA [13], among others [14].

Both nutritional and noncaloric compounds in breastmilk vary with time, evolving with the lactation stage. Interestingly, they also vary over the course of the day, displaying circadian rhythms. Thus, energy, nutrient, and bioactive compound intake are aligned with feeding/fasting cycles and synchronized with metabolic changes in breastfed infants. One of these micronutrients, melatonin, has several properties that should explain, at least in part, the protective role of breastmilk on further cardiovascular risk. In this review, we discuss the suggested mechanisms by which melatonin intake in early nutrition can program future cardiovascular health.

### 1.2. Melatonin: The Circadian Hormone

Melatonin, or N-acetyl-5-methoxytryptamine, is an indolamine derived from the amino acid tryptophan that is synthesized by the mammalian pineal gland in response to dark–light cycles [15]. Environmental light is sensed by the inner retina (retinal ganglion cells), which sends neural signals not only to the visual areas of the brain but also to the pineal gland through complex neuronal connections. Melatonin is synthesized every evening in the pineal gland in response to the decrease in light. Under darkness, glutamate is secreted, enhancing the transmission of the signal to a region of the hypothalamus, termed the “master clock,” which is located in the suprachiasmatic nucleus that orchestrates the cellular circadian mechanism. This neural center stimulates the superior cervical ganglion to release norepinephrine. Through adrenergic receptor activation, norepinephrine increases the levels of cAMP and the activation of the associated protein kinase A in pinealocytes [16]. The activation of this pathway is essential to melatonin production because it allows for an increase in the concentration of the enzyme arylalkylamine N-acetyltransferase, the limiting molecular step for melatonin synthesis in the pineal gland [15]. After biosynthesis, through blood flow, the resulting melatonin reaches all biological fluids and organs and synchronizes the peripheral clocks [17]. When the light signal is positive in the diurnal state, gamma-amino butyric acid is secreted, leading to the inhibition of the neuronal synapses in the nucleus of the hypothalamus; consequently, the signal is not received by the pineal gland, and melatonin is not synthesized. This complex regulation allows for metabolic pathways to be coordinated all over the organism and for the individual to be adapted to the 24 h cycle on Earth. The integration between the cyclic environment and the circadian regulation of physiological processes is thus provided, and the control of all endogenous rhythms at the tissue, cell, and subcellular levels is assured. In this way, endogenous melatonin induces the maintenance of stability and health in the organism and the hemostasis of diverse systems, such as the immune system and cardiovascular system, amongst others [18].

In addition to its action as the primary circadian hormone, melatonin is also a powerful antioxidant that is produced by all mitochondria-containing cells. As such, melatonin is a key regulator of immune defenses and cellular energy processes. There are particularly high concentrations of placental trophoblasts, immune cells, and enterochromaffin cells. In fact, following a meal, gut melatonin synthesis can be 400-fold that of the pineal gland’s night-time peak release [19].

Melatonin and circadian rhythms according to seasonal variation throughout the year can play a role in human reproduction similar to other mammals. It has been found that melatonin improves male fertility and progesterone production in the luteal phase, as well as oocyte maturation [18].

During the specific situation of pregnancy, melatonin is a powerful regulator of the mother–placenta–fetus interface and has a significant role in prenatal development. Fetal circadian rhythms develop through rhythmic maternal secretion of the hormones melatonin and cortisol. Over the course of development, the circadian oscillations of melatonin levels substantially increase and reach their peak before birth [16]. Simultaneously, extrapineal melatonin production in the placenta rises, and this feto-maternal connection drives the development of the fetal circadian system until birth and prepares the newborn for birth and the postnatal environment [20]. The increase in melatonin levels in the mother from 32 weeks of gestation underlines the importance of melatonin during the last trimester [21]. The circadian rhythm of the fetal heart rate is evident by 30 weeks of gestation, and by 32 weeks, different types of sleep can be distinguished [22]. To some degree, the loss of placental melatonin after birth can be compensated for by the presence of melatonin in breast milk. Maternal milk contains melatonin, which exhibits circadian oscillations, with lower levels during the day and higher levels at night, reaching a peak of approximately 40 pg/mL [23]. As the endogenous rhythm of melatonin production is not attained until approximately three months after birth and continues to develop over the first six months of life, the importance of melatonin intake through breast milk in early life should be emphasized [24,25,26]. Thus, during the first months of life, the newborn circadian machinery is developing, and breast milk constitutes the solely external source of melatonin. This property has special importance in preterm newborns, a population particularly at risk of future cardiovascular disorders [27]. These newborns lack the last part of the pregnancy that will provide the highest melatonin levels. Premature infants also have a delay in the rhythmic expression of melatonin with respect to full-term infants. However, the milk of mothers of preterm infants shows melatonin variation with a greater amplitude and higher concentration at the peak [28], suggesting that breast milk can be a tool for providing the melatonin that the child could not receive because of a preterm birth.

Either by endogenous synthesis or by exogenous intake, melatonin in blood is primarily transported by albumin to reach the peripheral organs. Its amphiphilic nature allows it to be present at the cell surface but also to reach the cytosol by diffusing through cell membranes or passing through transporters such as GLUT/SLC2A and PEPT1/2 [29,30].

**Figure 1:**
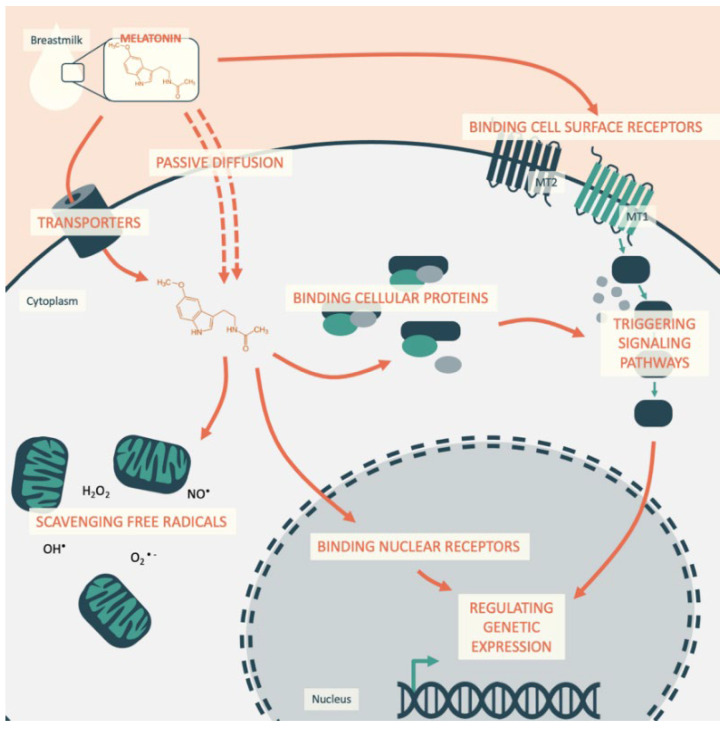
Melatonin cellular pathways in cardiomyocytes physiology.

In this way, melatonin acts on cellular homeostasis by: (1) binding to cell surface receptors; (2) reaching and binding nuclear receptors inside the cell; (3) binding cytosolic proteins; and (4) scavenging free radicals inside the cell (Figure 1).

1. When present at the surface of a cell, melatonin binds to specific G protein-coupled receptors named MT1 and MT2 [31], which are 350 and 363 amino acids long, respectively, and they are associated with proteins Gi and Gq. Melatonin then regulates the activity of adenylate cyclase, phospholipases C and A2, potassium and calcium channels, and guanylyl cyclase. These receptors are both present in the cardiovascular system, in which MT1 has been observed to mediate vascular constriction and MT2 vasodilatation. Their repartition changes in the vascular tree, explaining why melatonin has been reported as a vasoconstrictor in caudal arteries but a vasorelaxant in the aorta and mesenteric artery [31]. Melatonin also has an action on autophagy, which has been shown to be mediated by the α-7 nicotinic receptors [32].
2. Another pathway of action of melatonin occurs through reaching and binding nuclear receptors inside the cell and regulating transcription. The subfamilies of the nuclear receptors retinoid Z receptor/retinoid receptor-related orphan receptor, RZRa, RORa, RORa2, and RZRb reportedly have their activity regulated by melatonin, modulating the expression of their target genes [31]. For instance, following melatonin stimulation, RZR/RORa has been shown to bind DNA, increasing the transcription of the mRNA coding for ɣ-glutamylcysteine synthetase (ɣ-GCS), which is the limiting enzyme of glutathione (GSH) synthesis. Consequently, the enzyme and GSH are expressed to protect the cell from oxidative stress and to regulate the cell cycle [33]. It is believed that through these membrane and nuclear receptors, melatonin triggers clock gene expression by the direct circadian regulation of cellular pathways in both nucleated and non-nucleated cells. Melatonin also synchronizes the “peripheral clocks,” circadian oscillators present in all nucleated cells that ensure the presence of circadian rhythms of the organism even in the absence of an external synchronizer. They are composed of a group of genes known as “clock genes,” the circadian locomotor output cycles kaput (Clock), brain and muscle ARNT-like protein-1 (Bmal1), Period (Per), and Cryptochrome (Cry) genes, together forming a multiple loop of regulation with a period of approximately twenty-four hours. Under their control are “clock-controlled genes,” an important proportion of the genes involved in metabolism and inflammation, such as peroxisome proliferator-activated receptor γ (PPARγ), chemokine (C-C motif) ligand 2 (Ccl2), hypoxia-inducible factor (HIF)-1α, or glucose transporter 1 (Glut-1) [34]. For example, in adipose tissue, melatonin regulates the differentiation of adipocytes and the expression of clock genes (Clock, Per1) that increase the expression of their mRNA at night. In this way, melatonin also entrains the circadian rhythms of lipogenesis and lipolysis and fatty acid and glucose uptake [35,36,37].
3. In addition to regulating signals via receptors, melatonin activity is mediated by low-affinity interactions with proteins, such as enzymes, transporters, calcium-binding proteins, and cytoskeletal and scaffold proteins, both in the cytosol and organelles such as the mitochondria. Through its interaction with the Ca+-calmodulin complex, melatonin influences the formation of microtubules and has also been associated with endothelial nitric oxide synthase (eNOS) inhibition, triggering vasoconstriction [38]. Furthermore, by acting indirectly via its modulation of cellular oxidative stress, melatonin has been reported to regulate cellular pathways involving extracellular signal-regulated kinases (ERK1/2), protein kinase B (Akt), protein kinase C (PKC), and NFkB activation, leading to the regulation of cell proliferation [39].
4. Lastly, due to its biochemical structure, melatonin has the capacity to scavenge free radicals and limit oxidative stress inside cells [40].

Oxidative stress and inflammation are tightly interconnected and stimulate each other. The direct result of both is harmful to proteins, lipids, DNA, and cell organelles. Inflammatory cells produce numerous cytokines and chemokines that generate reactive oxygen and nitrogen species in both phagocytic and nonphagocytic cells by activating protein kinase signaling [41]. In fact, reactive oxygen and nitrogen species can trigger proinflammatory gene expression, and immune cells produce reactive species in response, leading to oxidative stress development. Melatonin is known to regulate the expression of cytokines and cyclooxygenase, in addition to nitric oxide synthetase activity and inflammasome activation. This activity is proposed to pass through the inhibition of nuclear factor kappa-light-chain-enhancer of activated B cells (NF-κB) translocation to the nucleus by modulating oxidative stress or the redox status and by directly interacting with proteins in the cytosol.

Understanding how circadian coordination by melatonin during early life then influences future cardiovascular health requires an assessment of the melatonin interaction at different levels. This indoleamine is involved in a series of biological functions and has important anti-inflammatory and antioxidant protective effects. In addition to endogenous melatonin production, the consumption of foods rich in melatonin significantly increases circulating melatonin levels in the range of physiological concentrations. However, breastfeeding is the sole source of melatonin during the first months of life, and the infant is dependent on this intake.

In this paper, we focus the importance of melatonin on several aspects regarding cardiovascular health. First, circadian hormones play a role because of the importance of circadian rhythms in cardiovascular health. Second, melatonin has a powerful antioxidant capacity and immunoregulatory effects. Lastly, it should also be emphasized that melatonin has the capacity to shape and interact with gut microbiota during this strategic moment of development.

## 2. Melatonin in Circadian Rhythms, in Relation to Cardiovascular Health

### 2.1. Melatonin and Circadian Rhythms

The composition of breast milk changes dramatically over a 24 h period and provides a powerful form of “chrononutrition.” It represents an important stage for the development of circadian rhythms with potentially significant effects not only on infant sleep but also on metabolism, growth, and future health.

Having a circadian rhythm implies a nocturnal secretion of melatonin that circulates through body fluids, including the milk of breastfeeding mothers [17]. Melatonin passes through the newborn digestive tract, interacts with the microbiota, and is absorbed by the intestine. Then, melatonin diffuses into the fluids of the newborn. This sequence of events is corroborated by the periodic presence of melatonin in the urine of breastfed but not formula-fed infants [42].

Melatonin that is periodically expressed for 24 h regulates and synchronizes a variety of reactions inside the cells in a circadian manner [43]. Regarding the cardiovascular system, peripheral clocks are present in each of the cardiovascular cell types, and clock genes and clock-controlled genes have an important role in cardiovascular homeostasis [44]. In the heart, approximately 8 to 10% of the genes display circadian variations. Consequently, the metabolism and activity of cells, such as cardiomyocytes, the endocrine system, and the autonomic nervous system, which are involved in the regulation of cardiovascular function, all display circadian rhythms [45,46]. The blood pressure shows daily variations with a particular surge at the end of the night at the time of waking [47]. It is also notable that the heart rate increases during the day and decreases during the night. Contrarily, coagulation activity increases during the night, is high in the morning, and decreases during the daytime. The vascular tone of the coronary artery, which decreases the coronary blood flow, also displays circadian rhythms with higher levels in the morning [48,49].

After birth, the heart and vascular system of the newborn continues to develop and grow. Cells such as cardiac fibroblasts continue to proliferate until being present in equivalent numbers as the cardiomyocytes in the adult heart [50]. These particular fibroblasts transmit the electric signal to adjacent cells, including cardiomyocytes, and are therefore involved not only in the structure but also in the function of the heart [50]. Moreover, the cellular microenvironment influences the proliferation, mobilization, and differentiation processes of progenitor cells involved in the growth and development of the cardiovascular system [50]. This sequence of events makes the first months of life a critical period for potential cardiac health programming by environmental factors [51].

The intake of melatonin through breast milk by newborns helps maintain their physiological mechanisms in a circadian manner. Through its direct activity on cellular pathways and via the synchronization of the peripheral clocks, melatonin entrains rhythms in the cardiovascular system that are essential to its homeostasis and function and, hence, to future cardiovascular health.

### 2.2. Circadian Rhythms and Cardiovascular Disorders

Melatonin and circadian rhythms play an essential role in cardiovascular health because they are involved in several cardiovascular pathologies and metabolic disorders [52]. Circadian rhythmicity is found in cardiac reactions to stress and in sensitivity to drugs [49,53,54,55]. These conditions can also explain why cardiovascular events tend to occur at a specific time of the day [46,48,56]. For instance, myocardial infarction, sudden cardiac death, and ischemic stroke tend to occur in the early morning [46,57].

In this sense, circadian rhythm impairment, which is known as chronodisruption, is a risk factor for cardiovascular events. This situation occurs when there is a misalignment of external stimuli (day–night cycle) and the inner clocks that oppose and desynchronize these intrinsic clocks with the metabolism. Night-shift work constitutes the paradigm of chronodisruption. As broadly studied in cohorts of nurses, an impairment in melatonin, such as the delay or suppression of its expression, is associated with increased weight, fat mass, and other cardiovascular risk factors [58,59,60]. Circadian rhythm impairment has been found to favor increases in blood pressure and interleukin-6 (IL-6), C-reactive protein, and tumor necrosis factor-α (TNF-α) levels, and to induce the dysregulation of glucose metabolism [61,62,63,64]. In animals, chronodisruption reduces longevity due to the appearance of cardiovascular events. Additionally, recovery after a cardiovascular event was slower in subjects with circadian disruption [57,58]. Sleep impairment is a known factor involved in the emergence of cardiovascular risk later in life [65]. Sleep deprivation is responsible for changes in cardiac autonomic control in healthy infants [66,67,68]. The impact of circadian rhythms on cardiovascular development and the associated programming of disease is also reflected by the increased tendency to have hypertension in the adult offspring of pregnant mothers with chronodisruption due to night-shift work [21].

Therefore, melatonin, with its potential action on the regulation of circadian rhythms, has a beneficial role in early nutrition. The melatonin absorbed by the infant through breast milk has been associated with an earlier entrainment in circadian rhythm as measured by actigraphy and with improved sleep duration and efficiency [69]. Breastfeeding has been associated with a reduced tendency toward irritability and colic and a longer sleep duration [70]. Another clue regarding the impact of breast milk melatonin on cardiac health lies in the counteraction of the delay in rhythmic melatonin expression observed in preterm children. This delay may affect cardiovascular development, explaining the increased predisposition to cardiac disorders in this population [71].

**Figure 2:**
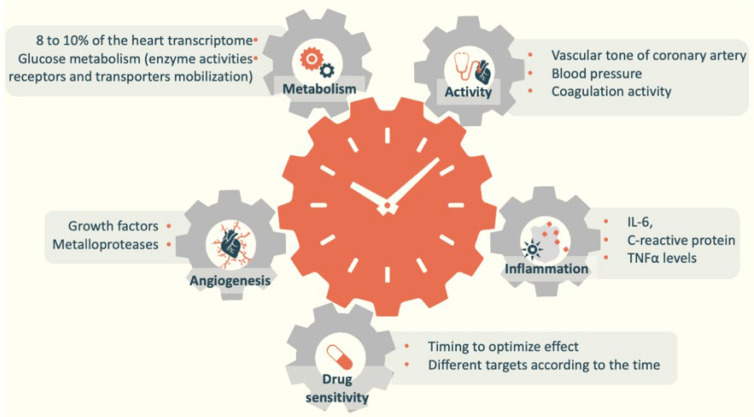
Essential parameters of cardiovascular physiology displaying circadian rhythms.

These elements underlie the participation of breast milk melatonin in newborn circadian rhythm entrainment and its contribution to shaping the cellular environment in a periodic manner (Figure 2). The available information on the importance of circadian rhythms in development, early life, and future health programming has led healthcare professionals to develop a chronobiological approach, highlighting the importance of this aspect of breastfeeding and ensuring a light environment adapted to neonatal physiology [26,72]. The positive outcome of breastfeeding on future cardiovascular events may be mediated by the influence on sleep and cardiovascular development. Taken together, these elements underlie the contribution of melatonin intake to the protective effect of breastfeeding.

## 3. Melatonin in Oxidative Stress and Inflammation in Relation to Cardiovascular Health

### 3.1. Melatonin in Oxidative Stress and Inflammation

In addition to its capacity to synchronize circadian rhythms, melatonin is known to be a powerful antioxidant that is involved in regulating inflammation [73,74,75]. Having antioxidant capacity implies actions through three pathways: (1) a molecular structure that acts as a scavenger of free radicals; (2) the stimulation of antioxidant enzymes; and (3) the inhibition of oxidant enzymes [76].

1. The structure of melatonin confers great scavenging capacity. The core is an electron-rich indole heterocycle that has high resonance. This core is the primary agent of the antioxidant action but is not the only one. The methoxy and amide side chains and the carbonyl group of the N-C = O structure in the C3 amide side chain provide melatonin with the capacity to scavenge more free radicals. This cascade is a biochemical characteristic of the melatonin molecule that other antioxidants, such as vitamin C, E, or glutathione, do not possess [77].
2. Furthermore, the biochemical structure is not the only way melatonin participates in reducing oxidative stress in the cell. In fact, the antioxidant capacity of melatonin also lies in the stimulation of enzymes involved in cellular antioxidant activity. This was evidenced by its stimulation of the genetic expression and enzymatic activities of glutathione peroxidase, glutathione reductase, superoxide dismutase, and catalase. Through glutathione reductase stimulation, melatonin helps to ensure a high GSH:GSSG ratio [78]. Additionally, at least in some human cells, melatonin can increase the ɣ-glutamyl-cysteine synthetase expression, allowing the de novo expression of glutathione [33].
3. Melatonin is involved in a third pathway that regulates oxidation by limiting the production of oxidants in the cell. It regulates the expression of the inducible nitric oxide synthase (iNOS). By lowering the expression of this enzyme, melatonin can indirectly prevent oxidative and nitrosative damage [38,79,80].

In addition to the direct modulation of cellular physiology by limiting oxidative stress and inflammation, there is another pathway through which melatonin may program future health: epigenetic modifications. By modifying the methylation of the DNA molecule around a specific gene or through histone acetylation processes, melatonin makes a change in the structure of the chromatin that will interfere with its capacity to be transcribed. The epigenetic mechanisms are reversible, transmissible, and adaptative and are regulated in part by the oxidative status of the cells. In fact, reactive oxygen species can interact with transcription factors and modify DNA methyltransferase and histone functions. They can promote alterations in DNA with subsequent methylation or interactions with the histone structure that result in changes in genetic expression. Therefore, molecules with powerful antioxidant capacities, such as melatonin, can protect against these changes and their long-term consequences [81]. By regulating oxidation, melatonin can indirectly regulate methylation. Melatonin has been shown to act via its MT1 receptor and the NFkB pathway, resulting in the hypermethylation of its receptor MT1 promotor and histone acetylation [82].

Therefore, melatonin is a high-performing antioxidant in vivo and can act as an epigenetic modifier. The intake through breastfeeding seems to be important for the infant.

### 3.2. Oxidative Stress, Inflammation, and Cardiovascular Health

The cardiovascular complications associated with obesity are also driven by processes involving hormones and peptides, and they include inflammation, insulin resistance, endothelial dysfunction, coronary calcification, activation of coagulation, renin angiotensin, or the sympathetic nervous systems.

Oxidative stress and inflammation form a self-perpetuating cycle that leads to cardiovascular disease pathogenesis and development. Oxidative stress causes damage to cells and tissues in the vascular system, inducing inflammatory reactions. In turn, inflammation causes an increase in reactive oxygen species and, therefore, increases oxidative stress. Thus, both oxidation and inflammation influence the physiology, function, and structure of the cardiovascular system, but they are also involved in the pathogenesis of cardiovascular diseases [83]. For instance, oxidized lipids generated by free radical-underregulated levels contribute to every step of atherosclerosis. In fact, some interventions are proposed to counteract these deleterious factors [84].

The oxidative and inflammatory status are key parameters of the cell microenvironment, not only through their levels but also through their variations over time. During pregnancy, cardiovascular development is tightly regulated by oxidant levels. In vitro, melatonin was found to regulate angiogenesis through the levels of growth factors, metalloproteases, and inflammatory parameters involved in the development of the vascular system [85].

Birth constitutes a great change in the fetal environment. In the mother’s womb, the fetus is in an environment with reduced oxygen and is surrounded by numerous antioxidants, including melatonin, ensuring the tight regulation of the oxidation status. At birth, when separated from the placenta, the fetus arrives in an environment that is richer in oxygen. As a result, oxidative stress increases, as does the role of antioxidants, including melatonin, in cardiovascular development.

In this context, melatonin, which has important anti-inflammatory and antioxidant protective effects, is a relevant candidate to consider for preventing cardiovascular diseases. It can modify the risk of obesity and related comorbidities because it is involved in biological functions including sleep regulation, blood pressure control, and the modulation of metabolic processes [86,87,88]. Melatonin was shown to interfere with a broad spectrum of molecular pathways. Recently, it has been shown that obesity-related cardiac hypertrophy is associated with an increase in mitochondrial oxidative stress and inflammation, an action mediated by sirtuins. Sirtuins are enzymes that act as histone deacetylases that interfere with the epigenetic environment of genes and consequently regulate their expression. More specifically, Sirt1 is closely involved in controlling the biological process of oxidative stress and has been shown to mediate the positive impact of melatonin intake on cardiac health [86,89].

## 4. Melatonin in Oxidative Stress and Inflammation in Relation to Cardiovascular Health

### 4.1. Melatonin in Gut Microbiota Regulation

In recent years, the understanding of the role of the gut microbiota has greatly evolved, leading to the consideration of the ensemble of symbiotic bacteria, viruses, and yeasts living inside the gut as an endocrine organ [90]. Numerous interactions occur between the host and the microbiota with participation in immunity, metabolism, and nervous activity [91]. The nutritional intake in terms of quantity, quality, diversity, and timing are determinant factors of microbiota development [92,93]. Intestinal motility is another important factor that can vary with the stress, sleep, mental status, or physical activity of the host.

The characteristics of the intestinal mucosa types of mucins and local and general immune factors are also factors involved in the microbiota composition. The physiological gut microbiota is dominated by Bacteroidetes and Firmicutes. A balanced gut microbial composition is critical to the host physiology, and compositional disruption has been associated with many diseases. For example, obesity is associated with a significant reduction in Bacteroidetes and a corresponding increase in Firmicutes [94]. Increased gut permeability and alterations in gut microbiota can drive immune-inflammatory processes that are linked to the etiology and pathogenesis of neurodegenerative disorders, depression, and other psychiatric problems [95].

During early life, two primary elements condition the first colonization of the digestive tract of the newborn: the type of delivery (vaginal or cesarean section delivery) and early nutrition (breast milk or milk formula) [96]. At birth, the infant has its first contact with microbes through the mother’s vaginal and skin microbiota. The early gut microbiome during the first days of life in infants who are born vaginally shares features with the vaginal microbial community and is dominated by *Prevotella* spp. and *Lactobacillus*, while, in infants born by cesarean section, the early gut microbiome resembles that of the maternal skin with *Corynebacterium*, *Staphylococcus*, and *Propionibacterium* spp. During the first weeks of life, the dominance of Actinobacteria (primarily comprising the genus Bifidobacterium) has been observed in vaginally delivered infants, while Firmicutes has been observed as the most prevalent microbial population for cesarean section-born infants. Moreover, the prevalence of Bifidobacteria continuously increased in both vaginally delivered and cesarean section infants over time. As the microbiota of infants born vaginally is associated with healthier incomes, some techniques have been developed to confer this protective effect to children born by cesarean section. The so-called “vaginal microbiota transfer” or “vaginal seeding” refers to the use of cotton gauze or cotton swabs with vaginal fluids to transfer the vaginal flora to the mouth, nose, or skin of a newborn infant. Through this procedure, the microbiota of cesarean section-delivered babies resemble that of naturally delivered newborns [95,97,98].

Regarding the type of early nutrition, the nidation of quantitatively and qualitatively specific bacteria in the gut varies. Higher levels of Bifidobacteria and Lactobacilli are present in the microbiota of breastfed infants, particularly *B. longum*, *B. infantis*, and *B. breve*, which can reach up to 60–90% of the total fecal microbiota in parallel with lower levels of potential pathogens [96].

Several nutritional factors, such as fatty acids and other bioactive compounds, such as human milk oligosaccharides and lactoferrin, influence microbial colonization. In turn, other components of breast milk, including tryptophan and omega-3 polyunsaturated fatty acids, enhance the production of melatonin that has a role in shaping the gut infant microbiota.

Among the compounds in breast milk involved in the microbiota composition, melatonin is known for its role in two essential features: (1) under physiological conditions, the microbiota displays circadian rhythms; (2) the antioxidant capacity of melatonin plays a pivotal role in the gut physiology.

(1) An extensive number of gut bacterial genera and species, as well as the microbial community, exhibit oscillatory behavior. The circadian rhythms of the gut microbiota are a feature of the physiology of a healthy gut microbiota, presenting daily fluctuations in community populations observable by changes in the most frequently represented bacterial strains, as well as circadian fluctuations in bacterial function and metabolite expression [99]. A clear association has been observed between dysbiosis and alterations in the circadian rhythms of the gut microbiota induced by nutritional factors and host metabolic disorders. In high-fat diet-fed mice (HFD), antibiotic treatment disturbed the gut microbiota and promoted metabolic impairment that improves after melatonin administration [100,101]. The gut microbiota of humans with circadian disruption secondary to jet lag, when transplanted into germ-free mice, induced obesity and glucose intolerance, an effect that was not observed when inoculating microbiota from non-jet-lagged individuals [102]. Melatonin has the capacity to reestablish the balance of GI microbiota by promoting the growth of Alistipes and Bacteroides, which are beneficial bacteria in the GI tract [103]. Thus, melatonin significantly improved metabolic disturbances, restoring healthy microbiota and circadian rhythms. The rhythmic expression of melatonin reaching the lumen of the gastrointestinal tract participates in synchronizing the circadian rhythms of the gut microbiota. For example, the gut bacteria *Enterobacter aerogenes* was observed responding to melatonin from the pineal gland and from the gastrointestinal tract by increasing its swarming activity [104]. Melatonin has the ability to decrease the Firmicutes-to-Bacteroidetes ratio in rodents and increase the abundance of mucin-degrading bacteria Akkermansia, which is associated with healthy mucosa [105]. The consequent disruptions to microbiota-mediated functions, such as the decreased conjugation of bile acids or increased production of hydrogen sulfide and the resulting decreased production of butyrate, in turn affect substrate oxidation and energy regulation in the host. Thus, disturbances in microbiome rhythms may at least partially contribute to an increased risk of obesity and metabolic syndrome associated with insufficient sleep and circadian misalignment. It is therefore likely that the rhythmic melatonin levels in breast milk have this effect on the newborn’s symbionts when they do not yet produce their own rhythmic melatonin.
(2) In addition, the action of melatonin on the gut microbiota and its interaction with the host physiology may be mediated by another pathway: the regulation of oxidative stress/inflammation. Melatonin is secreted by enterochromaffin cells in high quantities. In fact, the melatonin generated in the gastrointestinal tract surpasses pineal melatonin by 10–100 fold. It was speculated that most of the circulatory melatonin during the day was derived from the gastrointestinal tract. Its potent antioxidant activity contributes to maintaining the integrity of the intestinal barrier [106]. This barrier is composed of physical constraints, such as polymers of mucins limiting access to the epithelium, and numerous factors from the immune system ensuring the protection of the barrier, displaying circadian rhythms under physiological conditions [92,93]. The primary mechanisms by which melatonin is involved in this action are to regulate the abundance of matrix metalloproteinases, regulate immunological damage by the activity of macrophages, decrease oxidative stress, inhibit the production of nitric oxide, suppress the activity of NF-kB, and decrease the level of cytokines that promote inflammation [73].

### 4.2. Gut Microbiota Regulation and Cardiovascular Health

The microbiota produces compounds that are absorbed by the host and have a regulatory role in several metabolic pathways. This is the case for uremic toxins, anthocyanins, short-chain fatty acids, trimethylamine-N-oxide, phytoestrogens, bile acids, and lipopolysaccharides [107,108]. These compounds act through a diversity of mechanisms, such as vascular smooth muscle cell and endothelial cell mitogen-activated protein kinases (MAPK) and NFκB signaling, leading to inflammatory gene expression and the endothelial cell adhesion of leukocytes, in addition to the regulation of angiogenesis, influencing cardiomyocyte growth regulation, or by modulating cardiac activity [107]. When metabolized by the gut microbiota, some nutrients, such as choline, phosphatidylcholine, L-carnitine, and betaine, lead to a microbial expression of trimethylamine. Consequently, hepatic flavin monooxygenases oxidize trimethylamine N-oxide, which inhibits reverse cholesterol transport and the accumulation of macrophage cholesterol, promoting atherosclerosis. Secondary bile acid and indoxyl sulfate are associated with heart failure [107,108,109].

Regarding development and health programming, studies on germ-free animals provide interesting clues regarding the role of microbiota in development. The mice growing under these conditions display numerous disorders, such as altered structure and function in the intestine, liver, and lungs and altered endocrine activity. They also present a reduced total volume of blood, a decreased cardiac output, and increased cholesterol levels, numbers of red blood cells, and hematocrit in the blood [110]. These alterations outline the role of the microbiota in cardiovascular development and physiology. Both dysbiosis in the gut microbiota and altered intestinal barriers have been associated with a number of pathologies, such as obesity, type 2 diabetes mellitus, atherosclerosis, hypertension, and heart failure [111]. The gut microbiota of patients with hypertension presents a characteristic decrease in specific bacterial strains, such as Prevotella and Klebsiella; furthermore, the transfer of microbiota from these individuals to germ-free mice induces an increase in their blood pressure, demonstrating the transferability of this feature through microbiota [112]. Another study on mice demonstrated that melatonin supplementation improved several characteristics in mice consuming a HFD through modification of their gut microbiota, such as maintaining the Firmicutes-to-Bacteroidetes ratio to a healthy level. The levels of Akkermansia, a bacterial strain associated with intestinal physiology and protection against obesity, were also increased by melatonin supplementation, which also resulted in the prevention of obesity development and white adipose tissue hypertrophy and improved glucose tolerance and insulin [105]. The number and diversity of connections between gut microbiota and cardiovascular health explain the relevance of tight regulation in the intestinal absorption of microbial compounds and of the microbiota itself in terms of strain diversity and composition. The microbiota, which is shaped by the breastmilk consumption, metabolizes tryptophan and its derivates such as melatonin, inducing a direct regulation of its bioavailability [113]. Furthermore, by its role in the digestive health and as a regulator of the permeability of the intestinal barrier, the gut microbiota is likely to have an impact on melatonin absorption.

In summary, early nutrition is one of the two main determinant features of microbiota development. It shapes microbiota in a manner that is observable during early life and remains long term. Through its numerous interactions with the host’s health, the microbiota influences cardiovascular physiology and development during the first days of life and cardiovascular physiology later in life. Melatonin, through its role in circadian rhythms as in antioxidant capacity, is likely to be one of the compounds conferring breast milk with the capacity to shape a healthy microbiota in newborns, with positive long-term effects on cardiovascular health. Although further studies are required to quantify it properly, the current knowledge suggests that melatonin in breast milk influences the gut microbiota in the short and long terms by modifying the circadian rhythm, and, thus, it influences cardiovascular health programming.

## 5. Conclusions

Melatonin is a bioactive molecule with numerous known capacities that could contribute to the benefits of breastfeeding on long-term cardiovascular health. According to its circadian pattern in breast milk, this hormone is susceptible to inducing the first circadian stimulation in the infant’s body at an age when their own rhythmic melatonin expression is not functioning yet. Given the importance of circadian rhythms in cardiovascular physiology, earlier entrainment, as promoted by melatonin through breast milk consumption, may favor healthy development. Furthermore, melatonin is also an amphiphilic antioxidant with the capacity to directly and indirectly lower oxidative stress and inflammation in newborns. This modulation in the cardiovascular microenvironment at this stage of development is likely to shape the cell fate and physiology. Lastly, the gut microbiota, which has all the characteristics of an endocrine organ, will be the first to be shaped by breast milk compounds, including melatonin. By influencing the microbiota composition, diversity, and rhythms in the long term, melatonin may indirectly trigger cardiovascular health through this action. In all these ways, melatonin from breast milk can contribute to regulating cardiovascular development, the entrainment of circadian rhythms and epigenetics influencing obesity, and the associated comorbidities primarily related to cardiovascular risk. The breast milk constitutes the ideal source of melatonin in early life, because it is provided at an optimal time and in an adequate quantity. In the short term, melatonin absorption can result in observable effects on the structure and function of the organs and, in the long term, in the prevention of cardiovascular risk.

## Data Availability

Not applicable.

## Abbreviations

RZR/RORa	Retinoid Z receptor/retinoid receptor-related orphan receptor a
ɣ-GCS	ɣ-glutamylcysteine synthetase
GSH	Glutathione
Clock	Circadian locomotor output cycles kaput
Bmal1	Brain and muscle ARNT-like protein-1
Per	Period
Cry	Cryptochrome
PPARγ	Peroxisome proliferator-activated receptor γ
Ccl2	Chemokine (C-C motif) ligand 2
(HIF)-1α	Hypoxia-inducible factor
Glut-1	Glucose transporter 1
IL-6	Interleukin-6
NF-κB	Nuclear factor kappa-light-chain-enhancer of activated B cells
eNOS	Endothelial nitric oxide synthase
ERK1/2	Extracellular signal-regulated kinases
Akt	Protein kinase B
PKC	Protein kinase C
iNOS	Inducible nitric oxide synthase
MAPK	Mitogen-activated protein kinases
HFD	High-fat diet
